# Osthole‐loaded N‐octyl‐O‐sulfonyl chitosan micelles (NSC‐OST) inhibits RANKL‐induced osteoclastogenesis and prevents ovariectomy‐induced bone loss in rats

**DOI:** 10.1111/jcmm.15064

**Published:** 2020-03-03

**Authors:** Lining Wang, Suyang Zheng, Guicheng Huang, Jie Sun, Yalan Pan, Yuhao Si, Pengcheng Tu, Guihua Xu, Yong Ma, Yang Guo

**Affiliations:** ^1^ Laboratory of New Techniques of Restoration & Reconstruction Institute of Traumatology & Orthopedics Nanjing University of Chinese Medicine Nanjing China; ^2^ Department of Traumatology and Orthopedics Affiliated Hospital of Nanjing University of Chinese Medicine Nanjing China; ^3^ TCM Nursing Intervention Laboratory of Chronic Disease Key Laboratory Nanjing University of Chinese Medicine Nanjing China

**Keywords:** homology of medicine and food, mutual promotion, NFATc1, NSC‐OST, osteoclasts, osteoporosis

## Abstract

Osthole (OST), a derivative of Fructus Cnidii, has been proved to have potential anti‐osteoporosis effects in our recent studies. However, its pharmacological effects are limited in the human body because of poor solubility and bioavailability. Under the guidance of the classical theory of Chinese medicine, Osthole‐loaded N‐octyl‐O‐sulfonyl chitosan micelles (NSC‐OST), which has not previously been reported in the literature, was synthesized in order to overcome the defects and obtain better efficacy. In this study, we found that NSC‐OST inhibited on the formation and resorption activity of osteoclasts through using a bone marrow macrophage (BMM)‐derived osteoclast culture system in vitro, rather than affecting the viability of cells. We also found that NSC‐OST inhibited osteoclast formation, hydroxyapatite resorption and RANKL‐induced osteoclast marker protein expression. In terms of mechanism, NSC‐OST suppressed the NFATc1 transcriptional activity and the activation of NF‐κB signalling pathway. In vivo, ovariectomized (OVX) rat models were established for further research. We found that NSC‐OST can attenuate bone loss in OVX rats through inhibiting osteoclastogenesis. Consistent with our hypothesis, NSC‐OST is more effective than OST in parts of the results. Taken together, our findings suggest that NSC‐OST can suppress RANKL‐induced osteoclastogenesis and prevents ovariectomy‐induced bone loss in rats and could be considered a safe and more effective anti‐osteoporosis drug than OST.

## 1 INTRODUCTION

Bone remodelling refers to the physiological process through which the morphology and density of bone tissue change as the biomechanical environment changes. It is mainly accomplished via osteoblast‐mediated bone formation and osteoclast (OC)‐mediated bone resorption.^1^ Postmenopausal osteoporosis (PMO) is a systemic skeletal disease caused by an imbalance between bone resorption and bone formation. It is characterized by decreased bone density and increased bone fragility.^2^ Oestrogen deficiency‐induced formation and hyperfunction of OCs has been proven to be one of the important pathological features of high‐turnover metabolic bone diseases such as PMO.^3^ Therefore, inhibiting the differentiation and function of OCs may be a promising potential therapeutic strategy for treatment of PMO.

OCs, which are multinucleated cells derived from the haematopoietic lineage, play an important role in ensuring normal bone growth and development and maintaining bone homoeostasis.^4^ The formation and differentiation of OCs are subjected to multifaceted regulation involving cytokines, transcription factors and osteoclast‐related genes. Two cytokines, macrophage colony‐stimulating factor (M‐CSF) and receptor activator of nuclear factor kappa‐Β ligand (RANKL), have been recognized as the key to OC differentiation.^5^ M‐CSF is a prerequisite for the existence and proliferation of osteoclast precursor cells. RANKL binds to its cell surface receptor RANK (receptor activator of nuclear factor κB), resulting in the recruitment of TNF receptor‐associated factor 6 (TRAF‐6). In addition, RANKL further induces the activation of a key transcriptional gene in osteoclastic differentiation, nuclear factor of activated T cells 1 (NFATc1), by activating the nuclear factor‐κB (NF‐κB) and other signalling pathways.^6^, ^7^, ^8^ Eventually, specific downstream genes related to osteoclasts, including cathepsin K (CTSK), tartrate‐resistant acid phosphatase (TRAP), matrix metalloproteinase 9 (MMP‐9) and c‐Fos, are activated.^9^, ^10^, ^11^

Many of the drugs currently available for osteoporosis have rather severe side effects.^12^ Therefore, some natural products that have been shown to have anti‐osteoporosis potential have attracted considerable attention.^13^, ^14^ Osthole (OST) is a major active ingredient of the traditional Chinese medicinal plant *Cnidium*. Modern pharmacological research has found that OST possesses anti‐inflammatory,^15^ liver‐protecting,^16^ anti‐tumour^17^ and senile dementia‐delaying activities.^18^ In recent years, the anti‐osteoporotic effect of OST has gradually been expounded. It has been found that OST stimulates osteoblast differentiation and bone formation via the β‐catenin‐bone morphogenetic protein (BMP) signalling pathway or the cyclic adenosine 3′,5′‐monophosphate (cAMP)/response element‐binding protein (CREB) signalling network.^19^, ^20^ Consistent with this stimulation, Zhao et al^21^ reported that OST inhibited OC production and prevented bone loss in an ovariectomized animal model of osteoporosis. Our previous study found that, on the one hand, OST stimulated the proliferation and differentiation of osteoblasts by activating the Wnt/β‐catenin signalling pathway and causing endoplasmic reticulum stress and that this stimulation eventually promoted new bone formation.^22^ On the other hand, OST induced the down‐regulation of related transcriptional factors such as NFATc1 by suppressing the NF‐κB signalling pathway, thereby inhibiting the differentiation and maturation of OCs.^23^ However, due to its insolubility in water and its poor absorbability, the bioavailability of OST is limited in the body. To overcome these issues, our research group employed the concepts of ‘mutual promotion’ and ‘homology of medicine and food’ in traditional Chinese medicine and synthesized NSC‐OST (osthole embedded in N‐alkyl‐O‐sulfonyl chitosan), a novel compound that has not previously been reported in the literature. In the present study, we attempted to evaluate the effect of NSC‐OST on RANKL‐induced OC formation and activity. In addition, we further examined the anti‐osteoporotic effect of NSC‐OST in vivo by establishing an ovariectomized rat model of osteoporosis.

## 2 MATERIALS AND METHODS

### 2.1 Experimental animals

Forty nulliparous female Sprague‐Dawley (SD) rats were provided by the Qinglong Mountain Animal Breeding Farm (Nanjing, China; Certificate of Conformity: SCXK (Su) 2012‐0008). The rats were 3 months of age, specific pathogen‐free (SPF) grade and weighed 200 ± 15 g. This research project was approved by the Experimental Animal Ethics Committee of Nanjing University of Chinese Medicine (Approval No.: ACU170804).

### 2.2 Reagents and instruments

NSC‐OST (purity > 99%) was synthesized and provided by professor Jianping Zhou (China Pharmaceutical University, Nanjing, China). Osthole (purity > 99.5%) and nilestriol were purchased from the China Pharmaceutical biological products verification Institute (Beijing, China). ELISA kits of rat bone gla protein (BGP), TRAP, bone alkaline phosphatase (B‐ALP) and β‐isomerized C‐terminal telopeptides (β‐CTX) of type I collagen were provided by Jiancheng Bioengineering Institute. The Cell Counting Kit‐8 (CCK‐8) was purchased from Dojindo Molecular Technology. Recombinant murine macrophage colony‐stimulating factor (M‐CSF) and receptor activator of nuclear factor‐κB ligand (RANKL) were obtained purchased from R&D systems. TRAP staining kit and cyclosporine A (CSA) were obtained from Sigma‐Aldrich. Osteo Assay Surface hydroxyapatite‐coated plates were obtained from Corning Life Science. The kit of luciferase assay was obtained by Promega. The alpha‐modified minimal essential medium (α‐MEN), foetal bovine serum (FBS) and penicillin/streptomycin were purchased from Gibco. Antibodies against NFATc1, c‐fos, CTSK, TRAP, IκB‐α, p‐IκB, NF‐Κb p65, Lamin B1 and β‐actin were purchased from Cell Signaling Technology. The RAW264.7 Cells,^24^ transfected with luciferase reporter constructs under control of NFATc1‐blinding promoter elements, was generously provided by Professor Jiake Xu (University of Western Australia, Nedlands, Australia).

### 2.3 Cell culture

Bone marrow cells (bone marrow‐derived macrophages, BMMs) were isolated from rat tibia and femur under aseptic conditions according to a stable separation method established by our group.^25^ After purification by density gradient centrifugation, the cells were cultured under standard conditions (37°C, 5% CO_2_) in Minimum Essential Medium α (α‐MEM) containing 25 ng/mL M‐CSF, 10% foetal bovine serum (FBS) and 1% penicillin/streptomycin. The medium was changed every 2 days. Passage 1‐3 BMMs were used in the present study.

### 2.4 Cell viability assay

BMMs were seeded in 96‐well plates at a density of 10^4^ cells per well. The culture medium was replaced with medium containing OST, NSC‐OST (the actual OST content was 10^−5^ mol/L), or 0.25% dimethyl sulfoxide (DMSO) (Solvent control group). To maintain normal cell growth, M‐CSF (20 ng/mL) was included in the medium for all groups of cells. The medium was changed every other day. Cell viability was examined after 8 hours, 24 hours, 48 hours, 5 days and 7 days of drug intervention using the Cell Counting Kit‐8 (CCK‐8) method. Specifically, 10 μL of CCK‐8 solution was added to each well of cells. After further incubation of the plate in an incubator at 37°C for 4 hours, the absorbance at 450 nm was measured using a microplate reader (Perkin Elmar) and then the cell viability was analysed according to formula as described.^26^

### 2.5 Osteoclastogenesis assay

BMMs were seeded in 48‐well plates at a density of 1.5 × 10^5^ cells per well. The cells were divided into a negative control group, a control group, an OST group (10^−5^ mol/L) and an NSC‐OST group (the actual OST content was the same as OST group). All of the groups except the negative control group were incubated in medium containing 25 ng/mL M‐CSF and 100 ng/mL RANKL. Half of the medium was replaced every other day. After 5 days of induction, the cells were stained using the TRAP staining kit in accordance with the manufacturer's instructions. The cells were then examined under an inverted microscope (Olympus CKX31) and the number of TRAP‐positive multinucleated cells was calculated.

### 2.6 Bone resorption assay

A bone resorption assay was performed to evaluate the function of osteoclasts. BMMs were seeded into Osteo Assay Surface hydroxyapatite‐coated 96‐well plate at a density of 2 × 10^4^ cells per well. The cells were divided into four groups and treated with different conditions as described above. Then, the cells were incubated in 5% CO_2_ at 37°C for 5 days, and half of the medium was replaced every other day. After 5 days, the cells were removed by 10% bleach solution and the relative bone absorption area was determined by analysis of microscopic images using Image Pro Plus 6.0 software.

### 2.7 Western blotting

Western blotting assays were performed to assess the expression of some Osteoclast‐specific protein and activation of NF‐κB signalling pathway. The conditions of induction and grouping of OCs were the same as described above. BMMs were cultured in 12‐well plates (6 × 10^5^ cells/well) with or without M‐CSF (25 ng/mL) and RANKL (100 ng/mL) in the presence of NSC‐OST. Total cellular protein was prepared by RIPA buffer. Fractionation from cytoplasm and nucleus was separated from the whole protein by NE‐PER Nuclear and Cytoplasmic Extraction Reagents (Pierce Biotechnology). Equal amounts of protein were quantified by BCA method. The extracts from the whole cell (30 μg), cytoplasm (20 μg) and nucleus (10 μg) were separated by sodium dodecyl sulphate‐polyacrylamide gel electrophoresis (SDS‐PAGE) and transferred to polyvinylidene fluoride (PVDF) membranes. The membranes were blocked by 5% skim milk for 2 hours, then incubated with primary antibodies overnight at 4°C, such as NFATc1, TRAP, c‐FOS, CTSK, IκB‐α, p‐IκB, NF‐Κb p65, Lamin B1 and β‐actin. Then subsequently, the membranes were incubated with the respectively secondary antibodies conjugated by horseradish Peroxidase (HRP) for 2 hours at room temperature. The protein bands on membranes were exposed with the ECL imaging system (GE Quant LAS4000mini).

### 2.8 Luciferase reporter gene assay

RAW264.7 cells, transfected stably with NFAT‐luc, were randomly seeded into the 96‐well plates with each well containing 10^4^ cells. The cells were pre‐treated with OST, NSC‐OST or CSA for half an hour respectively. Then, the cells were induced by RANKL at the concentration of 100 ng/mL for next 24 hours. Eventually, following the instructions of the manufacturer, the cells were lysed, and the luciferase activities were analysed with the Luciferase Assay System (Promega).

### 2.9 Animal model construction and drug administration

All rats were reared and managed at the Experimental Animal Center of Nanjing University of Chinese Medicine. Three SD rats were housed in each cage to ensure that they had sufficient space to permit free movement and activity. The rats were allowed free access to food and water. The animal room was maintained at a temperature of 24 ± 2°C and a humidity of 60 ± 2% with a 12‐hour light/12‐hour dark cycle.

The rats were randomly divided into five groups after the oophorectomy. The rats received drug treatments for 12 consecutive weeks and were weighed once a week after surgery. The grouping of the rats and the doses administered were as follows: (a) The sham‐operated group (Sham) received an equal volume of normal saline (0.5% sodium carboxymethylcellulose). (b) The model group (OVX) received an equal volume of normal saline (0.5% sodium carboxymethylcellulose). (c) The nilestriol group (NIL) received NIL once weekly at 1 mg/kg/wk. For the remainder of the experimental period, the group received intragastric administration of the solvent. (d) The osthole group (OST) received OST at a dose of 10 mg/kg/d. (e) The osthole embedded in N‐alkyl‐o‐sulfonyl chitosan group (NSC‐OST) received NSC‐OST at a dose of 100 mg/kg/d (the drug load of OST was 10%, and the actual OST content remained the same). After 12 weeks of administration of saline or drugs by gavage, the rats were anaesthetized. The blood, uterus, bilateral tibias and femurs of the rats were collected for further experimentation.

### 2.10 Enzyme‐linked immunosorbent assay (ELISA)

The collected blood samples were placed at 4°C for 3 hours and then subjected to low‐temperature centrifugation. The serum, which appeared as a pale yellow transparent liquid in the upper layer, was collected and stored at −20°C for future use. The expression levels of BGP, TRAP, B‐ALP and β‐CTX of type I collagen in the serum were measured using commercial kits according to the instruction.

### 2.11 Biomechanical assay

Prior to the assay, the right femoral specimens were removed from storage and returned to room temperature. The length of the femur (along the long axis of the bone shaft) was measured using a Vernier caliper, and its midpoint was determined. The specimens were immobilized on a biomechanical tester (MTS Acumen 3) using clamps, and the span was adjusted to 1.5 mm. The femoral specimens were bent at a loading rate of 0.01 mm/s until fracture occurred, which was considered the end‐point of the assay. The three parameters, namely, maximum load, maximum deflection and stiffness, were calculated based on the load‐displacement curve generated by the instrument.

### 2.12 Micro‐CT

The structure of trabecular bone of the femur was determined by micro‐CT (Skyscan 1176). According to the protocol reported in the previous literature,^27^ the CT scan parameters were set as follows: resolution 9 μm, voltage 65 kV and current 384 mA; a 1‐mm filter was used. After the scan was completed, a uniform region of interest (ROI), namely, a section of cancellous bone with a length of 3 mm located approximately 0.5 mm from the intercondylar fossa growth plate, was selected for each specimen and reconstructed using the system's built‐in CTAn 1.14 software. Subsequently, the CTAn modelling software was used to construct and analyse two‐dimensional and three‐dimensional images. The following morphometric parameters of distal femoral cancellous bone were analysed: bone volume fraction (BV/TV), three‐dimensional bone mineral density (3D BMD), trabecular bone thickness (Tb.Th), number of trabecular bone (Tb.N) and trabecular bone separation (Tb.Sp).

### 2.13 Histopathological assays

HE staining: After fixation in 4% paraformaldehyde for 24 hours, the left femur was decalcified by soaking in 10% ethylenediaminetetraacetic acid (EDTA) solution for 4‐6 weeks. Specimens were collected at approximately 1.5 cm from the distal end of the femur, dehydrated using a graded ethanol series, cleared with xylene solution, routinely embedded in paraffin, sectioned (thickness: 5 μm), routinely HE stained and mounted in neutral resin. The morphology of the bone tissues was examined under an inverted microscope (Olympus CKX31).

TRAP staining: TRAP dye solution was prepared according to the instructions included with the kit. Cleared paraffin sections were incubated in the dye solution (preheated to 37°C) for 1 hour in the dark, nuclear‐counterstained with haematoxylin, rinsed with distilled water and mounted in neutral resin. The sections were examined under a microscope (Olympus CKX31), and the images were collected. The images were analysed using Image Pro Plus 6.0 software to determine the number of OCs per unit area of trabecular bone (N. Oc/BS) and the ratio of OC surface to trabecular bone surface (Oc.S./BS).

### 2.14 Real‐time quantitative polymerase chain reaction (RT‐PCR)

RT‐PCR was performed to quantify the expression of TRAP, MMP‐9, CTSK, NFATc1. Equal amounts of tissue were collected from the left tibial plateaus of the rats in all groups. The total tissue RNA was extracted using the Trizol method. The concentration and purity of the RNA were examined using a UV spectrophotometer. The reverse transcription system and PCR amplification system were set up according to the instructions included in the kits. After the reaction was completed, extension and melting curve analysis were conducted. Primer sequences were GAPDH: forward: 5′‐AAGTTCAACGGCACAGTCAA‐3′, reverse: 5′‐TACTCAGCACCAGCATCACC‐3′; TRAP: forward: 5′‐CTCGGCACAAATTGCCTACT‐3, reverse: 5′‐CACGGTGTCCAGCATAAAGA‐3′; MMP‐9: forward: 5′‐GCCGACTTATGTGGTCTTCC‐3′, reverse: 5′‐GCTTCTCTCCCATCATCTGG‐3′; NFATc1: forward: 5′‐AGCCATCATCGACTGTGCTG‐3′, reverse: 5′‐GGATGTGGACTCGGAAGACC‐3′; CTSK: forward: 5′‐TAATGTGAACCATGCCGTGT‐3′, reverse: 5′‐CGAGCCAAGAGAACATAGCC‐3′; GAPDH served as an internal reference. The relative expression levels of the target genes were determined using the 2^−ΔΔCt^ method.^28^

### 2.15 Immunohistochemistry

The sections of femurs were rehydrated with distilled water and with phosphate‐buffered saline (PBS) for 3 minutes each after dewaxing and dehydration. Subsequently, the sections were incubated for 30 minutes in preheated blocking buffer at room temperature in the dark. Following the incubation, the sections were washed twice with PBS. To achieve antigen retrieval, the sections were heated in a microwave oven in citrate buffer for 4 minutes to boiling and then allowed to cool naturally to room temperature. After being washed three times with PBS, the sections were blocked with bovine serum albumin for 30 minutes in a 37°C incubator, incubated with primary antibodies of NFATc1, CTSK and c‐fos (1:300 diluted) at 4°C overnight. The sections were then incubated with secondary antibodies at 37°C for 1‐2 hours followed by three washes with PBS. The DAB colorimetric reaction was then performed. The sections were counterstained with haematoxylin for 30 seconds, rinsed with water for 15 minutes, dehydrated with a gradient of ethanol, cleared with xylene and mounted in neutral resin. The sections were then examined under a microscope (Olympus CKX31) and mean optical density were semi‐quantified by the Image Pro Plus 6.0 as described elsewhere.^29^

### 2.16 Statistical analysis

The data were statistically analysed using GraphPad Prism 6.0. All measurement data are expressed as the mean ± standard deviation ($\bar{x}$ ± s). Multigroup comparison was conducted using one‐way analysis of variance (ANOVA). Pairwise comparison between the groups was performed using the Student‐Newman‐Keuls (SNK) test. *P* < .05 was considered statistically significant.

## 3 RESULTS

### 3.1 Effect of NSC‐OST on the body weight and uterus weight in OVX rats

**Figure 1:**
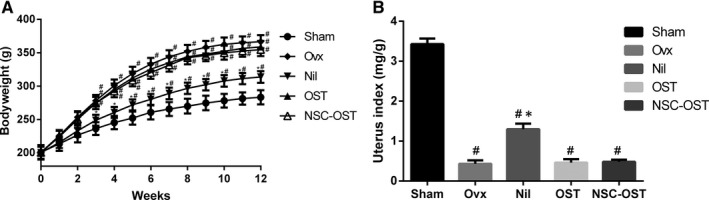
Effect of NSC‐OST on the body and uterus weight in OVX rats. (A) Bodyweight was measured once a week during the period of the experiment in the sham group, OVX group and OVX rats with oral administration of NIL at 1 mg/kg/wk or OST at 10 mg/kg/d or NSC‐OST at 100 mg/kg/d. (B) After killing the rats, uterus was isolated and weighted. The uterine index is the ratio of uterine weight to bodyweight. Dates are the Mean ± SEM (n = 8). #*P* < .05 vs Sham group, *P < .05 vs OVX group

As shown in Figure 1, the rats in the five experimental groups had similar initial weights. At 12 weeks after establishment of the model, all groups of rats experienced varying degrees of weight gain. The rats in the OVX group showed the most significant increase in body weight compared to the sham‐operated group, and all rats in the OVX group had significantly lower uterus indices than the animals in the sham‐operated group; the NIL group had a considerably elevated uterus index compared to the other three castrated groups.

### 3.2 Effect of NSC‐OST on the bone metabolism biochemical markers in OVX rats

**Figure 2:**
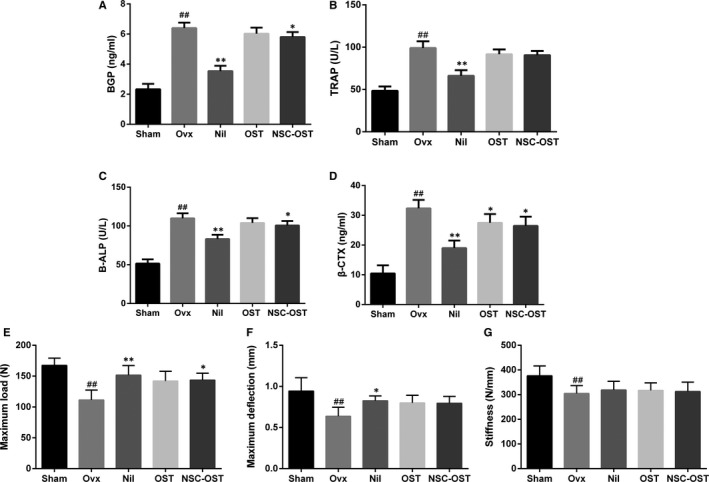
Effect of NSC‐OST on the bone metabolism biochemical markers and biomechanical parameters in OVX rats. (A) Bone gla protein (BGP); (B) tartrate‐resistant acid phosphatase (TRAP); (C) bone alkaline phosphatase (B‐ALP); (D) β‐isomerized C‐terminal telopeptides (β‐CTX) of type I collagen; (E) maximum load; (F) maximum deflection; (G) stiffness in each group was determined. Dates are the Mean ± SEM (n = 8). #*P* < .05 vs Sham group, ##*P* < .01 vs Sham group, *P < .05 vs OVX group, **P < .01 vs OVX group

Bone formation markers: After 12 weeks of drug administration, serum BGP and B‐ALP levels were significantly increased in the OVX group compared to the sham‐operated group. Compared with the model group, NIL significantly reduced the serum levels of BGP and B‐ALP. Although the serum levels of BGP and B‐ALP were reduced to a certain extent in the drug‐treated groups, only the NSC‐OST group showed markedly reduced serum BGP and B‐ALP levels compared with the model group (Figure 2A,C).

Bone resorption markers: After 12 weeks of drug administration, serum TRAP and β‐CTX levels were significantly elevated in the OVX group compared to the sham group. Compared with the model group, NIL significantly reduced the serum content of TRAP and β‐CTX. Drug treatment also inhibited the serum levels of TRAP and β‐CTX to some extent. However, there was no significant difference in the serum TRAP level between the two drug‐treated groups and the model group. In contrast, compared with the OVX group, both the OST and the NSC‐OST groups showed markedly reduced serum levels of β‐CTX (Figure 2B,D).

### 3.3 Effect of NSC‐OST on the biomechanical parameters in OVX rats

As shown in Figure 2E‐G, the maximum load, maximum deflection and stiffness of the femur were significantly inhibited in the OVX group compared to the sham‐operated group after 12 weeks of drug administration. Compared with the OVX group, treatment with NIL and NSC‐OST significantly enhanced the maximum load that could be applied to the femur before fracture occurred. In addition, treatment with NIL significantly increased the maximum deflection of rat femur in comparison with the OVX group. However, no significant differences were found in the stiffness of the femur between any of the drug‐treated groups and the OVX group.

### 3.4 Effect of NSC‐OST on bone mass in OVX rats

**Figure 3:**
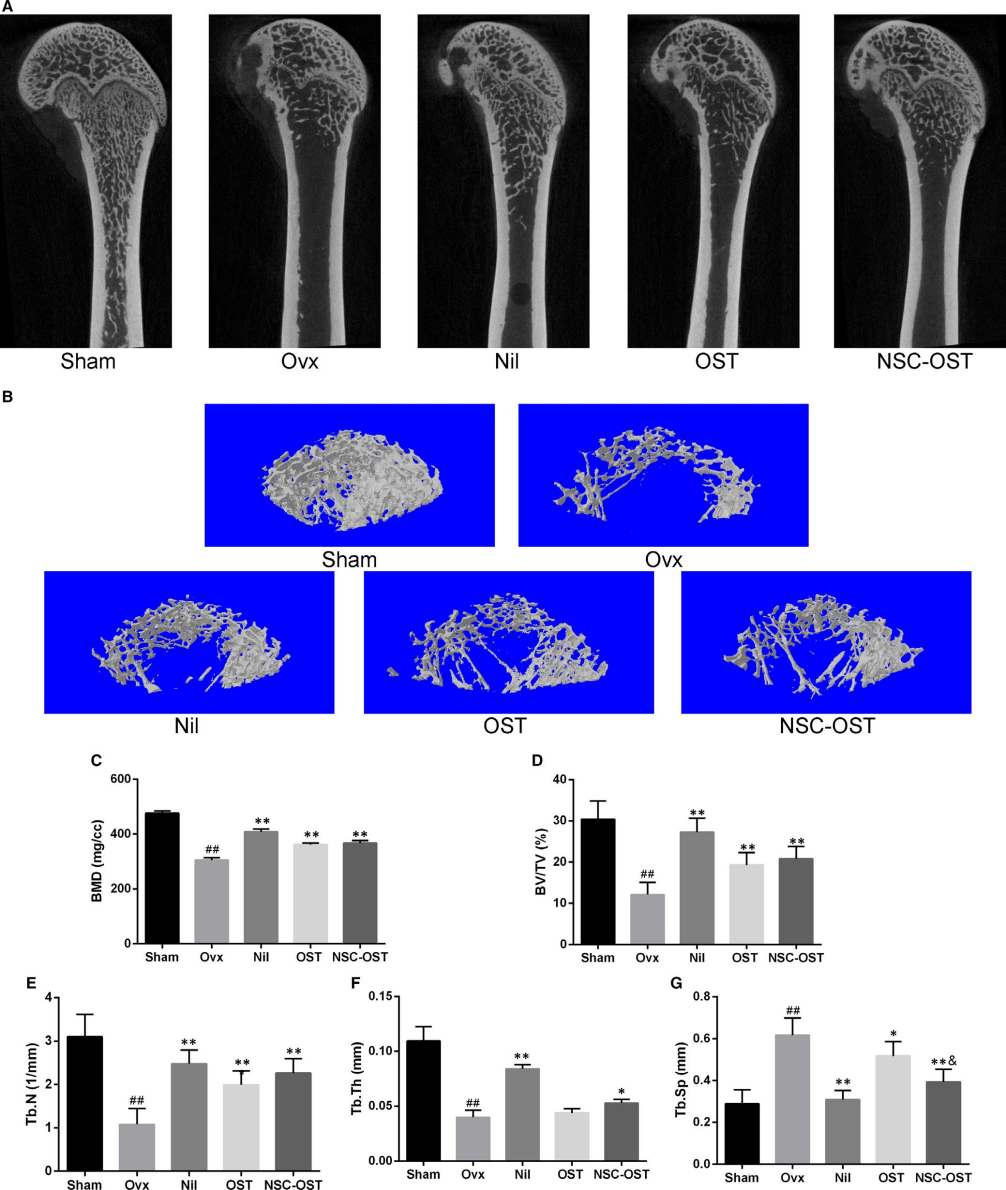
Effect of NSC‐OST on bone mass in OVX rats. (A) Representative 2D images of the vertical plane of the bone microstructure of the distal femur from the micro‐CT scans. (B) Micro‐CT 3D morphometry reconstruction of distal femur. (C) Bone mineral density (BMD); (D) bone volume/tissue volume (BV/TV); (E) trabecular number (Tb. N); (F) trabecular thickness (Tb. Th); (G) trabecular separation (Tb. Sp). Dates are the Mean ± SEM (n = 5). #*P* < .05 vs Sham group, ##*P* < .01 vs Sham group, *P < .05 vs OVX group, **P < .01 vs OVX group, &*P* < .05 vs OST group, &&*P* < .01 vs OST group

**Figure 4:**
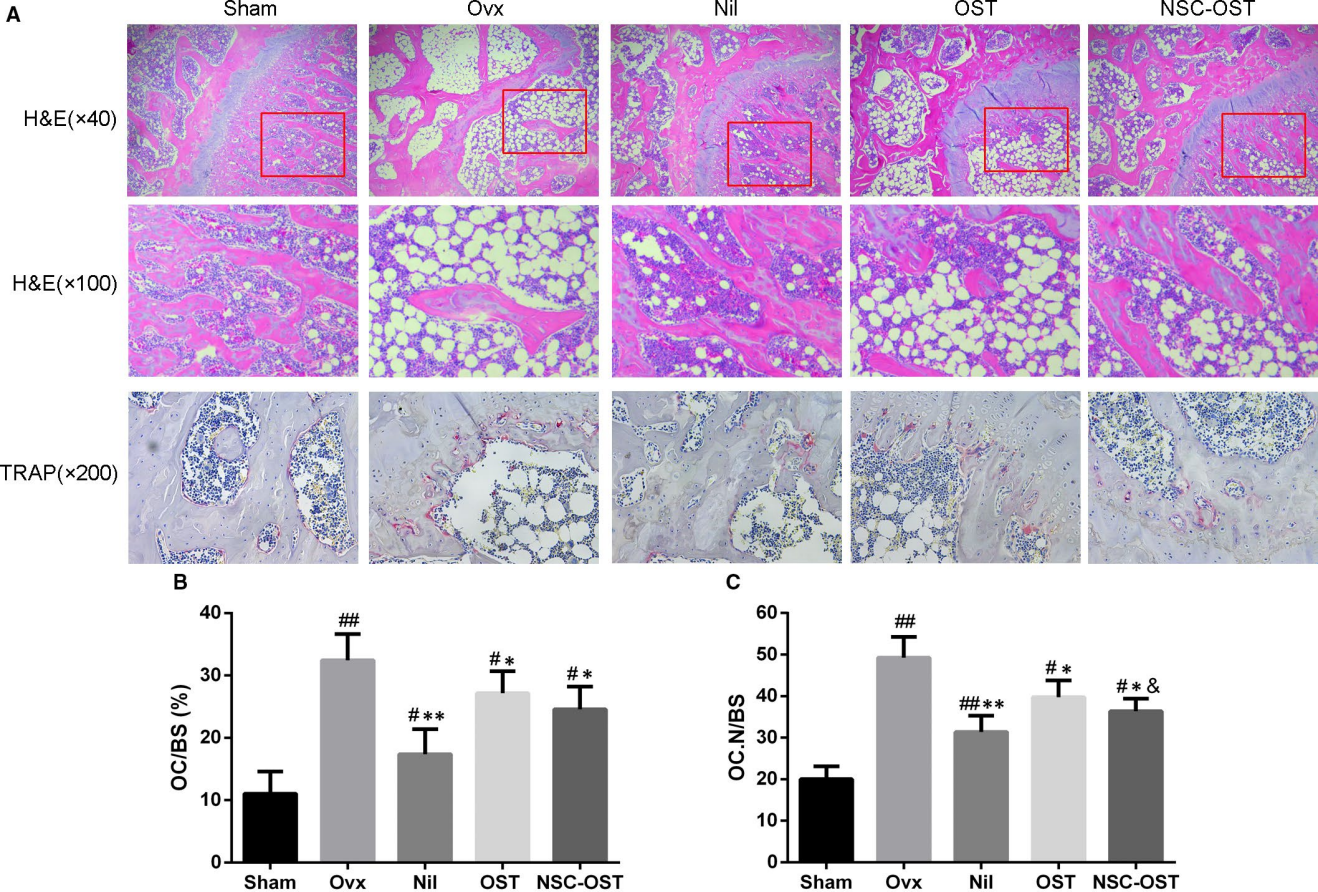
Effect of NSC‐OST on the histopathological changes of femurs in OVX rats. (A) Morphologies of cortical and trabecular bone tissue of proximal femur were examined by H&E staining. Original magnification was 40×. (B) Effect of OST on the formation of osteoclasts in vivo was examined by TRAP staining. Original magnification was 200×. (C) Osteoclast surface/bone surface (OC.s/BS) and (D) Osteoclast number/bone surface (OC.N/BS) ratios were analysed by Image Pro Plus 6.0 software. Dates are the Mean ± SEM (n = 3). #*P* < .05 vs Sham group, ##*P* < .01 vs Sham group, *P < .05 vs OVX group, **P < .01 vs OVX group, &*P* < .05 vs OST group, &&*P* < .01 vs OST group

To further investigate the therapeutic effect of NSC‐OST on bone mass in vitro, Micro‐CT scans and 3D reconstruction were used to evaluate the changes in bone microstructure in the rat model (Figure 3A,B). The model group exhibited significant trabecular bone loss compared with the sham‐operated group. However, compared with the model group, NSC‐OST significantly increased the BMD and BV/TV of the animals and significantly improved Tb.N, Tb.Sp and Tb.Th (Figure 3C‐G). HE staining further supported the following additional results (Figure 4A). The rats in the model group exhibited typical osteoporosis‐like changes. Specifically, the bone cortex and bone trabeculae became significantly thinner and showed loss of connectivity and widening of inter‐trabecular spaces. After 3 months of drug treatment, bone tissues in the NIL, OST and NSC‐OST groups were significantly improved and showed similar morphologies. Specifically, bone trabeculae in all three groups became slightly thinned, showed an orderly arrangement and formed web‐like structures.

### 3.5 Effect of NSC‐OST on osteoclast‐associated gene expression in OVX rats

**Figure 5:**
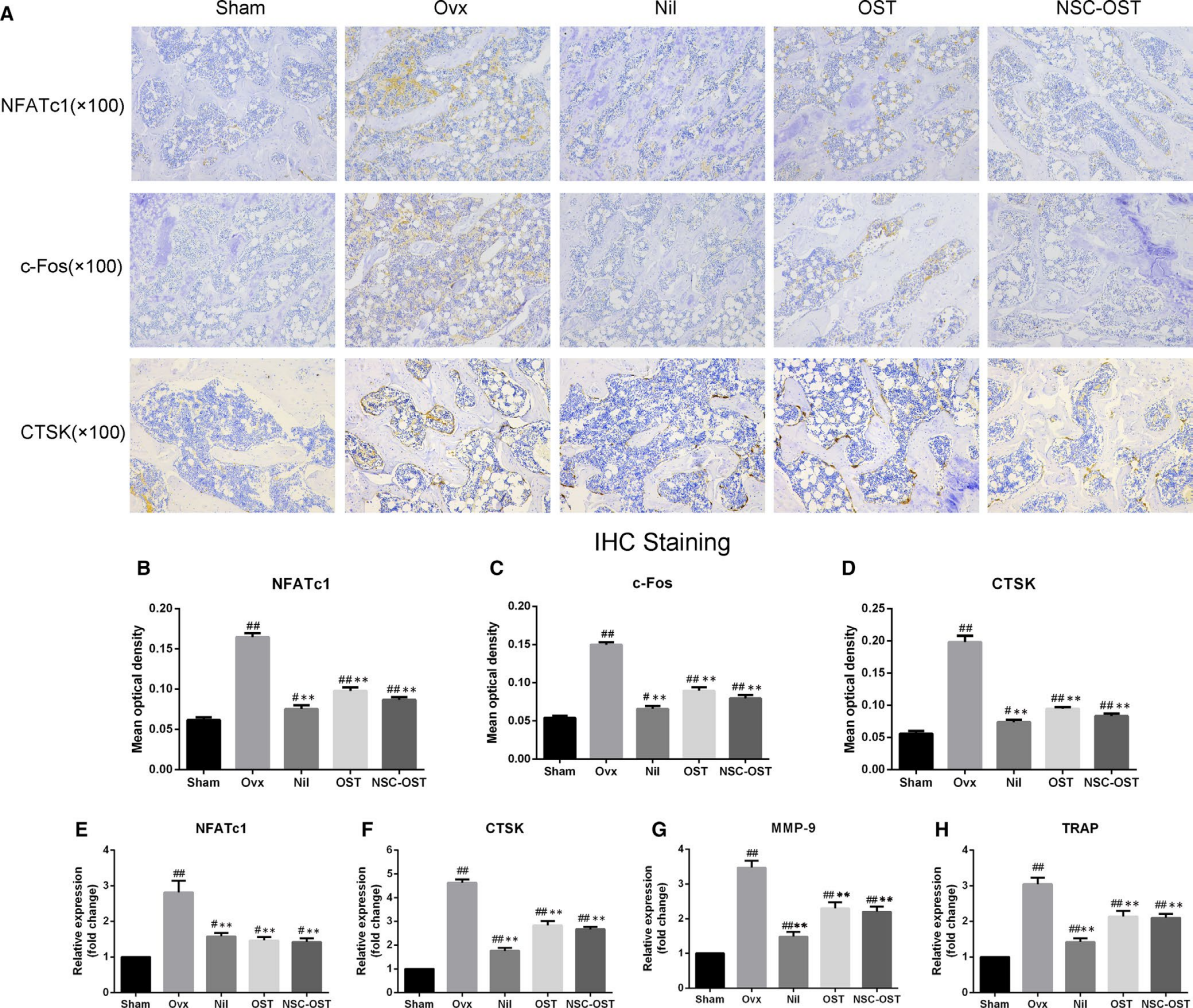
Effect of NSC‐OST on the expression of osteoclast‐specific markers in OVX rats. (A) The expression of NFATc1, c‐Fos and CTSK with tibia platform from rats in each group was detected by IHC staining. Original magnification was 100×. Semi‐quantitative analysis was performed for measuring the mean optical density of (B) NFATc1, (C) c‐Fos and (D) CTSK by Image Pro Plus 6.0 software. The osteoclast‐specific gene expressions of (E) NFATc1, (F) CTSK, (G) MMP‐9 and (H) TRAP with tibia platform from rats in each group were measured and analysed by the 2^−ΔΔCt^ method. Dates are the Mean ± SEM (n = 3). #*P* < .05 vs Sham group, ##*P* < .01 vs Sham group, *P < .05 vs OVX group, **P < .01 vs OVX group

As shown in the Figure 5E‐H, The mRNAs extracted from the cancellous bone of the tibial plateau of rats in the experimental groups were specifically amplified. The mRNA expression levels of NFATc1, CTSK, MMP‐9 and TRAP were significantly elevated in the OVX group compared with the sham‐operated group. Nilestriol intervention significantly inhibited the mRNA expression of these genes in comparison with the model group. Compared with the model group, the mRNA expression of NFATc1, CTSK, MMP‐9 and TRAP was also significantly inhibited after the intervention of NSC‐OST. However, there were no significant differences between the OST group and the NSC‐OST group in any of the genes examined.

### 3.6 Effect of NSC‐OST on BMMs viability

**Figure 6:**
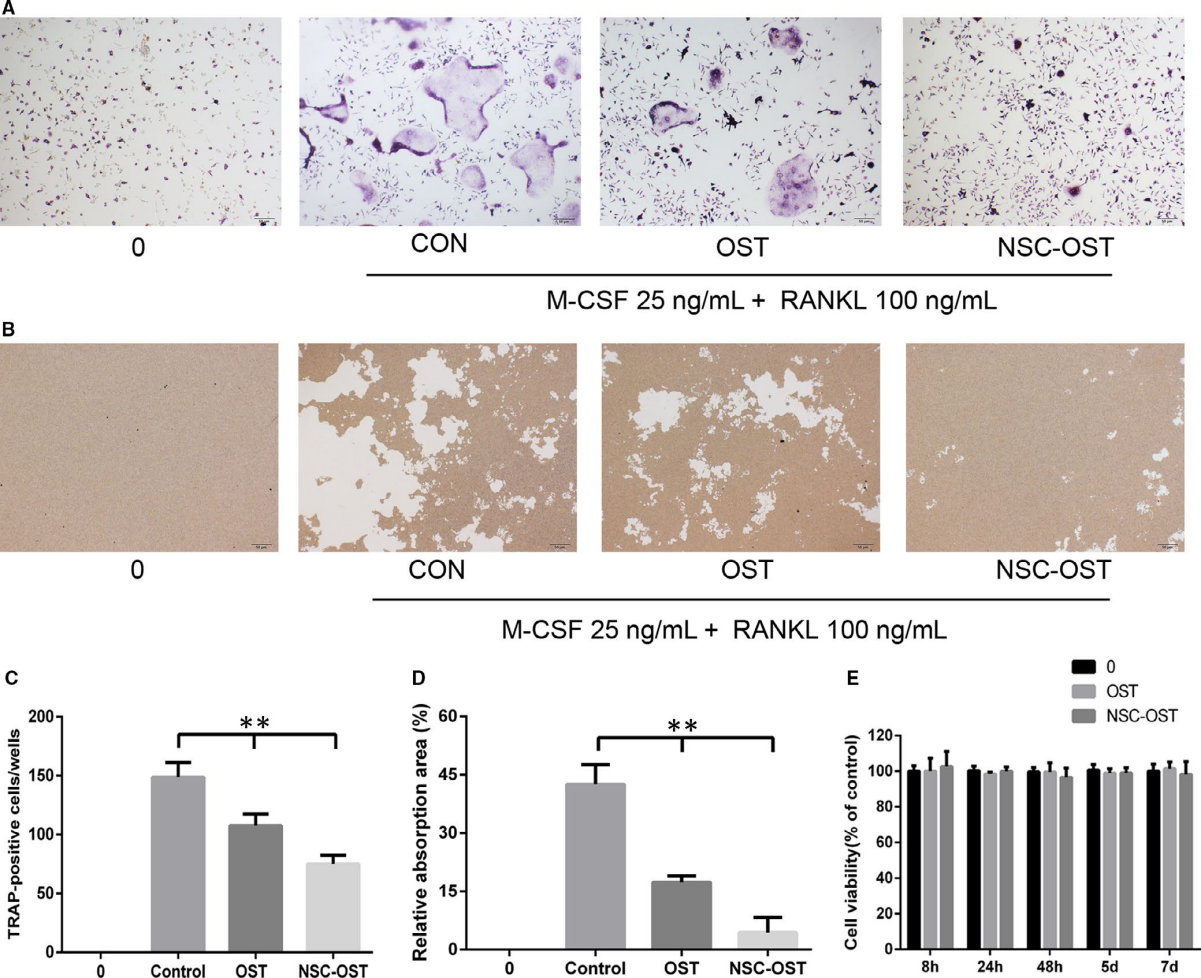
Effect of NSC‐OST on the osteoclastogenesis in vitro. (A) Representative TRAP staining images of RANKL‐induced osteoclast formation in different groups for 5 d, Original magnification was 40×. (B) Representative images of resorbing area in hydroxyapatite‐coated plates. Original magnification was 40×. (C) Quantification of TRAP‐positive multinucleated cells, five microscopic fields of view were randomly selected at a magnification of 40×, and the cells with ≥3 nuclei were enumerated. (D) Quantification of relative bone absorption area was analysis by Image Pro Plus 6.0 software in different groups. (E) Viability of BMMs cells treated by NSC‐OST with CCK‐8 method. Dates are the Mean ± SEM (n = 3), **P < .01

As shown in the Figure 6E, OST and NSC‐OST showed no significant inhibitory effect on cell proliferation at any of the time‐points examined. This finding suggests that NSC‐OST has no obvious cytotoxicity to BMMs.

### 3.7 Effect of OST on the formation and activity of osteoclasts in vitro and vivo

To evaluate the effect of NSC‐OST on RANKL‐induced osteoclastogenesis of BMMs, BMMs were treated with the same effective concentrations of OST or NSC‐OST in the presence of M‐CSF and RANKL. According to the results of TRAP staining, M‐CSF and RANKL effectively induced osteoclast formation and differentiation (Figure 6A,C). In contrast, both OST and NSC‐OST significantly inhibited the formation of TRAP‐positive multinucleated osteoclasts, and the inhibitory effect of NSC‐OST was more potent than that of OST.

To examine the effect of NSC‐OST on the bone resorption activity of osteoclasts, BMMs were cultured on the bone slices and treated with OST or NSC‐OST for 48 hours in the presence of M‐CSF or RANKL. The results of the area of bone resorption showed that both OST and NSC‐OST significantly inhibited the formation of bone resorption lacunae (Figure 6B,D). Moreover, the effect of NSC‐OST was more significant.

To further examine the effect of NSC‐OST on osteoclast formation in vivo, femoral sections of the rat model were subjected to TRAP staining. The staining results showed that the number of osteoclasts was significantly increased in the OVX group compared with the sham group. OST and NSC‐OST treatment significantly inhibited the formation of osteoclasts (Figure 4A), and the inhibitory effect of NSC‐OST was more significant than that of OST. Image analysis with Image J showed that N. Oc/BS and Oc. S/BS were significantly increased in the model group in comparison with the sham group. The drug treatments reversed the two parameters to varying degrees. The reversal effect of the NIL group was the most significant, followed by the NSC‐OST group and then the OST group (Figure 4B,C). The above results indicate that NSC‐OST significantly inhibited the formation and activity of osteoclasts both in vivo and in vitro.

### 3.8 Effect of NSC‐OST on the NFATc1 transcriptional activity and the expression of related protein in vitro and vivo

**Figure 7:**
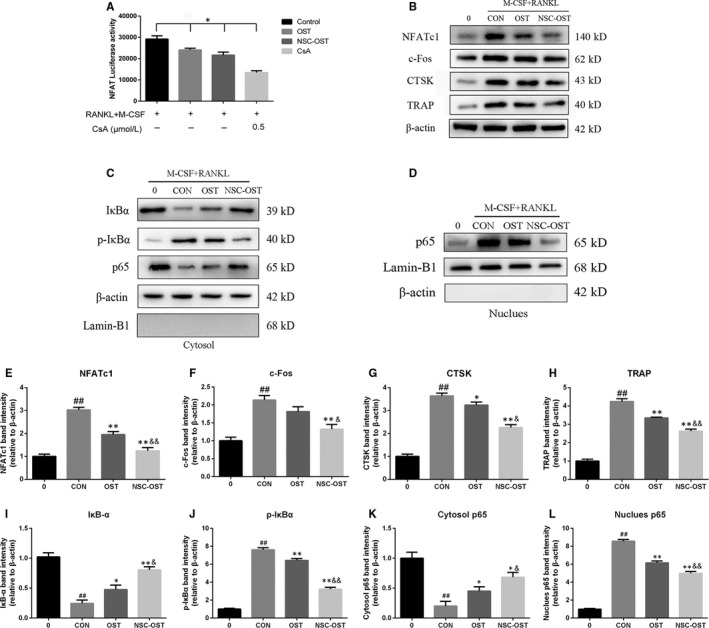
Effect of NSC‐OST on the NFATc1 transcriptional activity, osteoclast‐related protein expression and the activation NF‐κB pathway. (A) The stably transfected RAW264.7 cells were pre‐treated with OST or NSC‐OST and CsA for half an hour, then administrated with 100 ng/mL RANKL for 24 h. The results show that NSC‐OST suppresses the transcriptional activity of NFATc1 by the luciferase reporter gene assay. (B) BMMs were pre‐treated with OST or NSC‐OST for 1 h then offered 25 ng/mL M‐CSF and 100 ng/mL RANKL stimulation for 24 h. Afterwards, whole cell proteins were extracted, and the protein expressions of related proteins were measured by Western blotting analysis, including NFATc1, c‐Fos, CTSK and TRAP. In addition, proteins of NF‐κB signalling pathway from (C) cytoplasm and (D) nucleus were extracted and measured by Western blotting analysis separately. (E‐H) NFATc1‐related protein expression were analysed to those in control. (I‐L) NF‐κB‐related protein expression of IκB‐α, p‐iκB and NF‐κB p65 were analysed to those in control. Dates are the Mean ± SEM (n = 3). #*P* < .05 vs Sham group, ##*P* < .01 vs Sham group, *P < .05 vs CON group, **P < .01 vs CON group, &*P* < .05 vs OST group, &&*P* < .01 vs OST group

The RANKL‐induced transcriptional activity of NFATC1 was determined in the presence of NSC‐OST intervention. The results of luciferase reporter gene assays showed that NSC‐OST significantly inhibited RANKL‐induced NFATc1 transcriptional activity (Figure 7A). Consistent with this result, the NFATc1 and related downstream proteins such as c‐fos and CTSK during osteoclast differentiation was also reversed by OST and NSC‐OST. The effect of NST‐OST was more potent than that of OST (Figure 7B,E,F). Further, immunohistochemical assays and its results of semi‐quantitative analysis were performed to examine the expression of NFATc1 and related proteins in femoral sections, and similar results were obtained (Figure 5A‐D). These results suggested that NSC‐OST can inhibit the NFATc1 activity and the expression of related protein both in vitro and vivo.

### 3.9 Effect of NSC‐OST on RANKL‐induced NF‐κB signalling pathway in vitro

Intracellular cell signaling by induction of RANKL is the activation of NF‐κB pathway, which subsequently mediates NFATc1 activation. To determine the influence of NSC‐OST on the NF‐κB pathway. We used the proteins from cytoplasm and nucleus to detect the effect of NSC‐OST on the phosphorylation of IκB‐α and the translocation of p65 by Western blotting analysis. As seen in Figure 7C,D,I‐L, induced by M‐CSF an RANKL can increase a phosphorylation‐related IκB degradation, along with the nuclear translocation of p65. The results showed that NSC‐OST can suppress the degradation of IκB and the phosphorylation of p65 obviously and NSC‐OST has a more effective influence than OST on the inhibition of NF‐κB signalling pathway.

## 4 DISCUSSION

Compatibility of medicines is the soul of traditional Chinese medicine prescriptions, and compatibility of ‘mutual promotion’ is the most common mode of compatibility in compound prescriptions. Mutual‐promotion compatibility refers to the phenomenon in which combined administration of two drugs with similar performance and efficacy leads to the enhancement of single or multiple therapeutic effects. The prevention and treatment of osteoporosis in the traditional Chinese medicine emphasizes the ‘homology of medicine and food’ and often involves medicinal food ingredients derived from animal shells or bones. Chitosan is derived from chitin, the main component of fish, shrimp and crab shells. It has a wide range of sources and is able to form amphiphilic polymer micelles through structural modification. The polymer micelles can be used to physically embed drugs and solubilize insoluble drugs, thereby improving drug bioavailability.^30^ In addition, chitosan itself has bone formation‐promoting activity and has been effectively applied in bone tissue engineering.^31^ Based on the good prospects of OST against osteoporosis, our cooperative partner successfully prepared NSC‐OST with high hydrophilicity and further explored the pharmacological effects of NSC‐OST on the differentiation of OCs and in an ovariectomized rat model of osteoporosis in the present study.

Since we had focused on OCs in our previous study, we first examined whether NSC‐OST is capable of inhibiting osteoporotic bone loss through suppression of the formation and activation of OCs. It was first observed in the present study that OST and NSC‐OST are not cytotoxic to BMMS at an effective concentration of 10^−5^ mol/L. Moreover, both OST and NSC‐OST inhibited the formation and bone resorption activity of OCs, and NSC‐OST exhibited a more potent inhibitory effect. The effect of NSC‐OST on osteoblast‐mediated bone formation still needs to be further elucidated.

NFATc1 is an indispensable transcriptional factor in OC formation and differentiation.^32^ In addition, NFATc1 also plays an important role in the activation, fusion and bone resorption function of OCs.^33^ NFATc1‐deficient embryonic stem cells are unable to differentiate into OCs, whereas ectopic expression of NFATc1 causes osteoclast precursor cells to undergo efficient differentiation in the absence of RANKL. Targeted destruction of NFATc1 in mouse haematopoietic cells significantly reduces the number of OCs and increases bone mass.^34^ c‐Fos is not only an important upstream regulatory molecule of NFATc1 during OC differentiation but also an indispensable transcriptional factor downstream of RANKL.^35^, ^36^ c‐fos homozygous mice experience a series of OC deficiency‐induced dysfunctions.^37^ In addition, NFATc1 regulates the expression level of its target genes by directly binding to the promoter region, thereby ultimately regulating the formation and functioning of OCs. The target genes of NFATc1 include CTSK and MMP‐9, which affect the secretion of degrading enzymes by OCs and the bone resorption activity of OCs, as well as TRAP, a specific marker enzyme of OCs.^38^ The present study found that NSC‐OST reduced the expression levels of several OC‐specific mRNAs that are up‐regulated by RANKL. Moreover, the effect of NSC‐OST was superior to that of OST. These results indicate that NSC‐OST inhibits the formation and bone resorption activity of OCs by regulating the expression of the transcription factor NFATc1.

At present, the commonly studied cellular signalling pathways in OC differentiation include the NF‐κB, c‐Jun N‐terminal kinase (JNK), extracellular‐signal‐regulated kinase (ERK)/p38 signalling pathways and so on.^39^ Among them, NF‐κB pathway is one of the most important roles, which has been proved to be participated in various osteolysis disease.^40^ In the current study, these results suggest that NSC‐OST has more inhibitory effects than OST on osteoclast differentiation and function, which suppress osteoclast‐associated marker expression and that the activation of NF‐κB pathways are involved in the anti‐osteoclastogenic effects of NSC‐OST. The other signal transduction pathways through which NSC‐OST affects the differentiation of OCs are worth further exploration.

To further evaluate the therapeutic efficacy of NSC‐OST in vivo, an ovariectomized rat model of osteoporosis was established in the present study to simulate PMO. All of the rats in the OVX group showed significant atrophy of the uterus and rapid weight gain, indicating successful establishment of the model. It is worth noting that serum bone metabolism markers were significantly elevated in the OVX group. This increase is consistent with the characteristics of high‐turnover metabolic bone diseases. Oral administration of OST and NSC‐OST inhibited the increase in these markers, and the effects of NSC‐OST on BGP, B‐ALP and β‐CTX were more significant than those of OST. It is well known that osteoporosis is a chronic metabolic bone disease that is characterized by decreased bone mass and degeneration of the bone microstructure. Micro‐CT, HE staining and biomechanical assays have been widely used to assess bone mass and quality.^39^, ^41^ The results of all three types of assays showed that oral administration of NSC‐OST significantly inhibited bone loss in the OVX rats and improved bone microstructure to a certain extent. Moreover, the effect of NSC‐OST was superior to that of OST partially. The results of pathological TRAP staining of rat femur were consistent with the results of the in vitro experiments. It was found that the formation and activity of OCs were significantly inhibited by NSC‐OST.

In summary, this study demonstrated that NSC‐OST synthesized under the guidance of classical Chinese medicine theory inhibited the formation and activity of OCs and improved bone loss in OVX rats. Therefore, NSC‐OST could be considered a safe and effective anti‐osteoporosis drug. However, the amount of NSC‐OST available is only sufficient to meet the needs of the research project at present. Thus, the process of preparation of NSC‐OST needs to be improved for further researches.

## CONFLICT OF INTEREST

The authors declare no conflict of interest.

## AUTHOR CONTRIBUTIONS

Lining Wang, Yong Ma and Suyang Zheng conceived and designed the experiments. Lining Wang, Suyang Zheng and Jie Sun performed the experiments. Lining Wang, Yalan Pan, Pengcheng Tu and Yuhao Si analysed the data. Guicheng Huang, Yalan Pan, Guihua Xu and Yang Guo contributed to reagents/materials/analysis tools.

## Data Availability

The data sets used and/or analysed during the current study are available from the corresponding author on reasonable request.
